# Supplementary material for: Mechanistic insights into Alpha-Synuclein binding to P2RX7: A molecular dynamic and docking study
Source: PLoS One. 2025 May 2;20(5):e0319098. doi: 10.1371/journal.pone.0319098 (PMC12047839; doi:10.1371/journal.pone.0319098)
Supplement: S1 Fig — (A) SNCA (PDB ID 1XQ8) containing NTD (1-98aa) and CTD (99-140). The NTD possess Phospholipid binding region (1-60), Low affinity cholesterol binding region (32-43), Glycosphingolipid (GSL) binding region (34-45aa), high affinity cholesterol binding region (67-78) and hydrophobic core(61-95) comprising non-amyloid β-component (NAC). (B) P2RX7 (PDB ID 6U9W) comprises an external domain (ETD; 47-334 aa), two trans membrane domains (TMD1; 26-46 aa and TMD2; 335-355 aa) and an intracellular N- (1-25) and C-terminal (356-595) cytoplasmic domain (17). ETD further divided into head (111-169), upper body (70-92, 105-115 and 291-315), lower body (50-68, 94-106,188-209, 250-277 and 316-327), left flipper (278-292), right flipper (178-189, 235-250), and dorsal fin (206-234). ATP binding sites comprises K64, K66, T189, N292, R294 and K311 residues followed by subsidiary residues (R276, R277, K193, D280, Y288, L191, I214, I228, E186 and N187). The TMD consisting of TMD1 and TMD2 made up of α1 helix (24-48) and α6 helix (331-358) respectively. TMD is containing cytoplasmic cap (β-1, β0, and β15). The Cytoplasmic N-terminal comprises 1-25 amino acids whereas C-terminal domain (358-595aa) comprises cysteine rich regions (CRRs), hexagonal ~ 14 Å widened cytoplasmic pore formed by α12- α13, cytoplasmic plug (α9), large bulky cytoplasmic ballast (α9–α16 and β16–β1) harbouring lipids and GDP binding sites. (PDF) [file pone.0319098.s001.pdf]

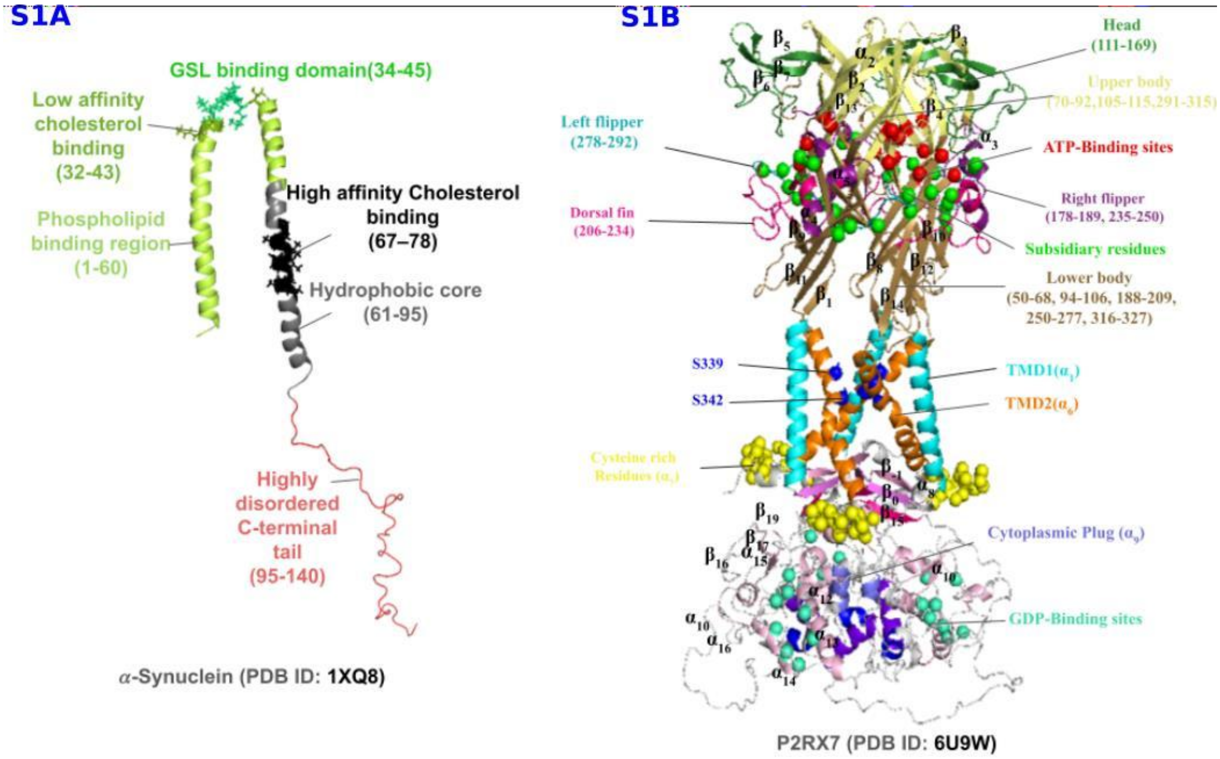


**S1Fig. Structural features annotation.**

(A) SNCA (PDB ID 1XQ8) containing NTD (1-98aa) and CTD (99-140). The NTD possess Phospholipid binding region (1-60), Low affinity cholesterol binding region (32-43), Glycosphingolipid (GSL) binding region (34-45aa), high affinity cholesterol binding region (67-78) and hydrophobic core(61-95) comprising non-amyloid β-component (NAC). (B) P2RX7 (PDB ID 6U9W) comprises an external domain (ETD; 47-334 aa), two trans membrane domains (TMD1; 26-46 aa and TMD2; 335-355 aa) and an intracellular N- (1-25) and C-terminal (356-595) cytoplasmic domain (17). ETD further divided into head (111-169), upper body (70-92, 105-115 and 291-315), lower body (50-68, 94-106,188-209, 250-277 and 316-327), left flipper (278-292), right flipper (178-189, 235-250), and dorsal fin (206-234). ATP binding sites comprises K64, K66, T189, N292, R294 and K311 residues followed by subsidiary residues (R276, R277, K193, D280, Y288, L191, I214, I228, E186 and N187). The TMD consisting of TMD1 and TMD2 made up of α1 helix (24-48) and $\alpha_6$ helix (331-358) respectively. TMD is containing cytoplasmic cap ($\beta_{-1}$, $\beta_0$, and $\beta_{15}$). The Cytoplasmic N-terminal comprises 1-25 amino acids whereas C-terminal domain (358-595aa) comprises cysteine rich regions (CRRs), hexagonal ~14 Å widened cytoplasmic pore formed by $\alpha_{12-}$ $\alpha_{13}$, cytoplasmic plug ($\alpha_9$), large bulky cytoplasmic ballast (α9–α16 and β16–β1) harbouring lipids and GDP binding sites.
