# Supplementary material for: Mechanistic insights into Alpha-Synuclein binding to P2RX7: A molecular dynamic and docking study
Source: PLoS One. 2025 May 2;20(5):e0319098. doi: 10.1371/journal.pone.0319098 (PMC12047839; doi:10.1371/journal.pone.0319098)
Supplement: S2 Fig — (A) Backbone RMSD of αSyn alone, P2RX7 alone and P2RX7-αSyn complex of all the open and close forms of P2RX7 with and without αSyn were estimated for 200 ns time frame. The higher RMSD of αSyn alone is the distinguished in the line graph as compared to apoP2RX7 and P2RX7-SNCA complexes. The topmost left and right upper panel shows RMSD of closed (hP2RX7-6U9V) and open forms (hP2RX7-6U9W) of apoP2RX7 as orange line graph. The left and right middle panel shows RMSD of SNCA alone (blue), hP2RX7 alone (orange) and hP2RX7-SNCA complex (green) in line graph of respective closed and open forms of P2RX7-SNCA complex of replicate-1(hP2RX7-6U9V-SNCA1 & hP2RX7-6U9W-SNCA1). Similarly, the left and right bottom panel shows RMSD of SNCA alone (blue), hP2RX7 alone (orange) and hP2RX7-SNCA complex (green) in line graph of respective closed and open forms of P2RX7-SNCA complex of replicate-2 (hP2RX7-6U9V-SNCA2 & hP2RX7-6U9W-SNCA2). (B) The radius of gyration (Rg) analysis reveals that αSyn alone is less compact compared to both apo P2RX7 and the P2RX7-SNCA complexes. Interestingly, apo P2RX7 exhibits a slightly higher Rg than the protein complexes, indicating a marginally more compact structure. The color scheme used aligns with that of the RMSD analysis. (C) RMSF of apoP2RX7 and P2RX7-SNCA complex models after 200 ns MD simulations. There are certain regions in P2RX7 in P2RX7 in both apo and complexes which are quite flexible. Most of these regions (Head, ATP binding sites, cytoplasmic ballast). The open form of P2RX7 (hP2RX7-6U9W) is depicted in aquamarine, while the closed form (hP2RX7-6U9V) is shown in dark orange. Their respective complexes are represented as follows: hP2RX7-6U9W-SNCA1 and hP2RX7-6U9W-SNCA2 in sky blue and pink, and hP2RX7-6U9V-SNCA1 and hP2RX7-6U9V-SNCA2 in khaki and green. (D) Distribution of first 10 eigenvectors of all the models after PCA analysis of 175 ns MD trajectories (25-200ns). First three eigenvectors of each model within the box show their proport [file pone.0319098.s002.pdf]

S2A

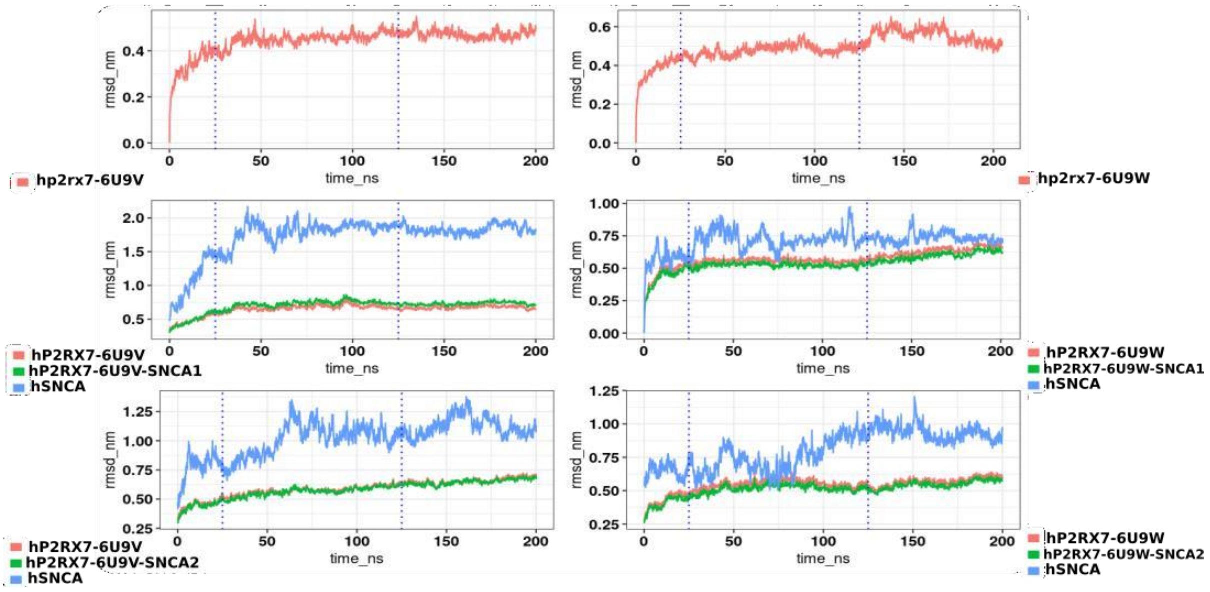

rmsd_nm
time_ns
hp2rx7-6U9V
hp2rx7-6U9W
hP2RX7-6U9V
hP2RX7-6U9V-SNCA1
hSNCA
hP2RX7-6U9W
hP2RX7-6U9W-SNCA1
hSNCA
hP2RX7-6U9V
hP2RX7-6U9V-SNCA2
hSNCA
hP2RX7-6U9W
hP2RX7-6U9W-SNCA2
hSNCA

S2B

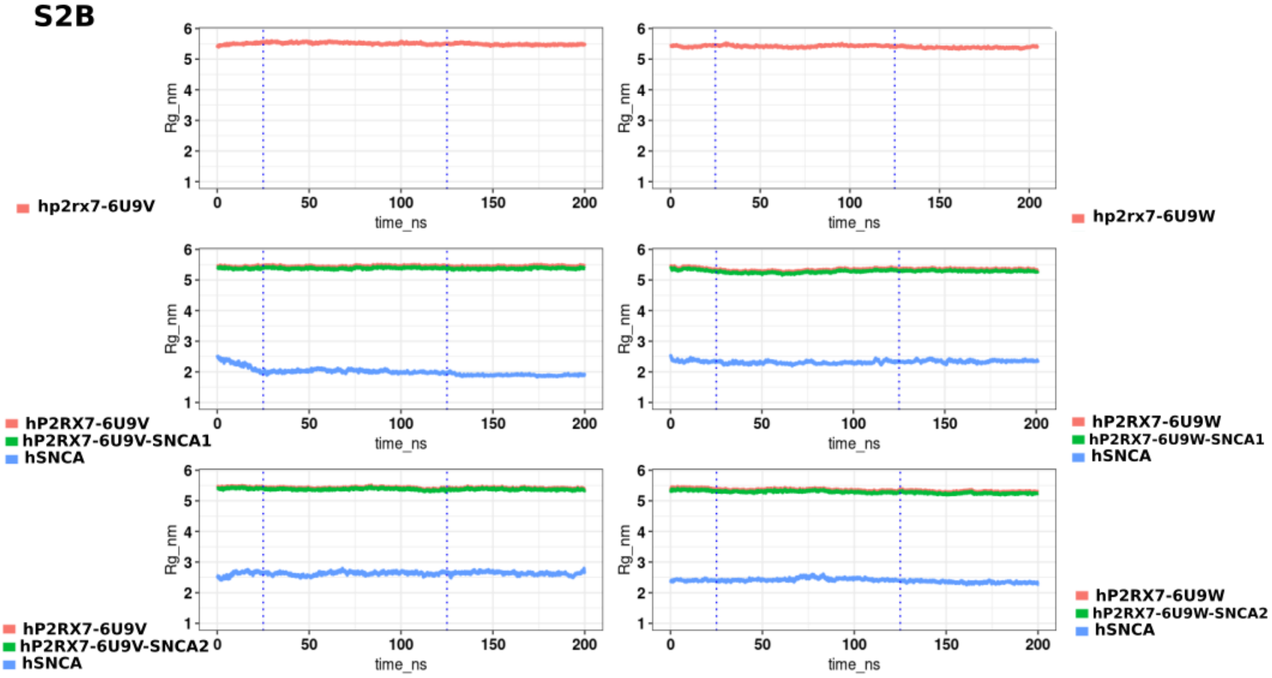

Rg_nm
time_ns
hp2rx7-6U9V
hp2rx7-6U9W
hP2RX7-6U9V
hP2RX7-6U9V-SNCA1
hSNCA
hP2RX7-6U9W
hP2RX7-6U9W-SNCA1
hSNCA
hP2RX7-6U9V
hP2RX7-6U9V-SNCA2
hSNCA
hP2RX7-6U9W
hP2RX7-6U9W-SNCA2
hSNCA

S2C

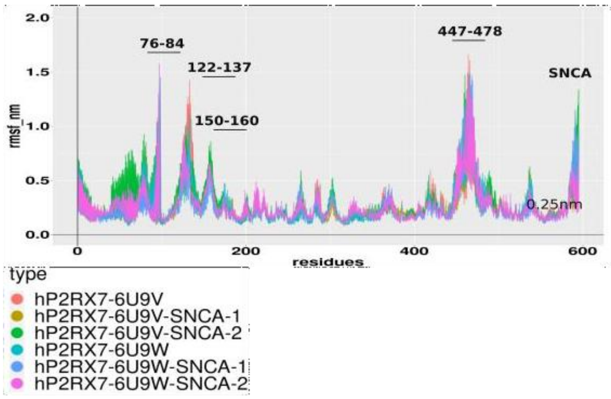

RMSF(in nM)
76-84
122-137
150-160
447-478
SNCA
0.25nm
rmsf_nm
2.0
1.5
1.0
0.5
0.0
0
200
400
600
residues
type
hP2RX7-6U9V
hP2RX7-6U9V-SNCA-1
hP2RX7-6U9V-SNCA-2
hP2RX7-6U9W
hP2RX7-6U9W-SNCA-1
hP2RX7-6U9W-SNCA-2

S2D

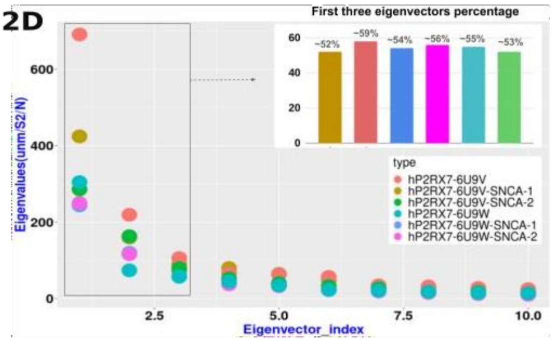

First three eigenvectors percentage
~52%
~59%
~54%
~56%
~55%
~53%
Eigenvalues(unm/S2/N)
Eigenvector_index
type
hP2RX7-6U9V
hP2RX7-6U9V-SNCA-1
hP2RX7-6U9V-SNCA-2
hP2RX7-6U9W
hP2RX7-6U9W-SNCA-1
hP2RX7-6U9W-SNCA-2

S2E

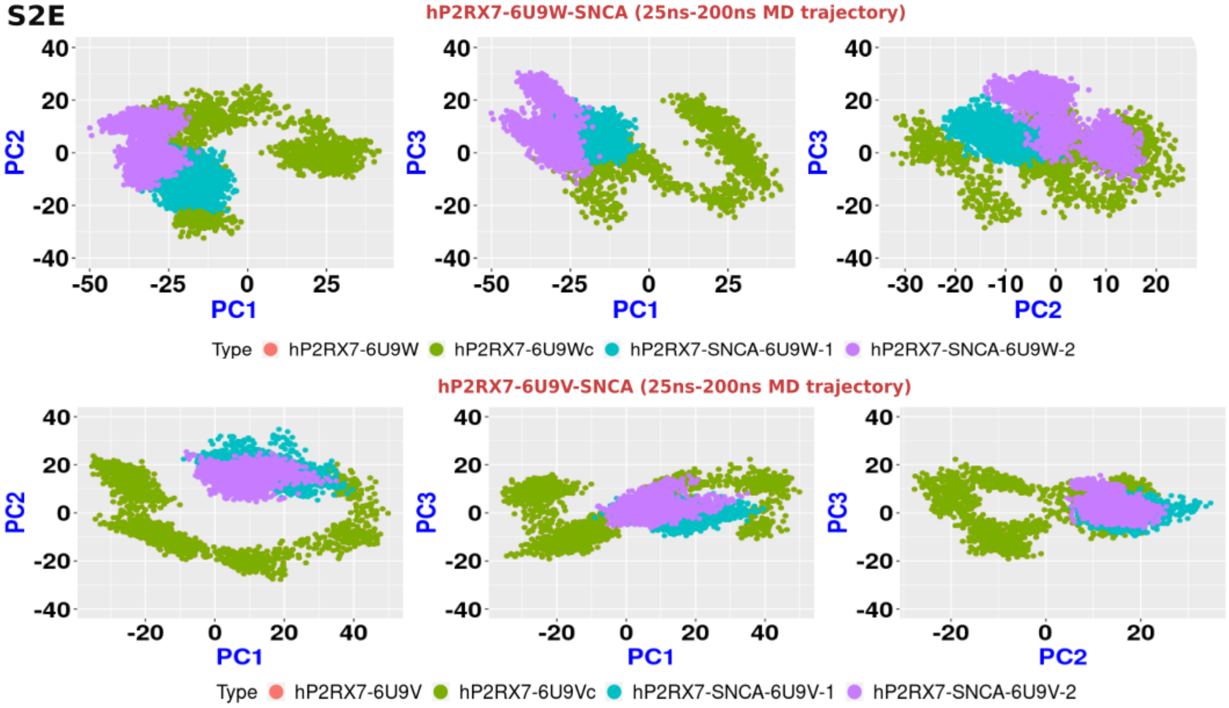

hP2RX7-6U9W-SNCA (25ns-200ns MD trajectory)
PC1
PC2
PC3
Type hP2RX7-6U9W hP2RX7-6U9Wc hP2RX7-SNCA-6U9W-1 hP2RX7-SNCA-6U9W-2
hP2RX7-6U9V-SNCA (25ns-200ns MD trajectory)
Type hP2RX7-6U9V hP2RX7-6U9Vc hP2RX7-SNCA-6U9V-1 hP2RX7-SNCA-6U9V-2

S2F

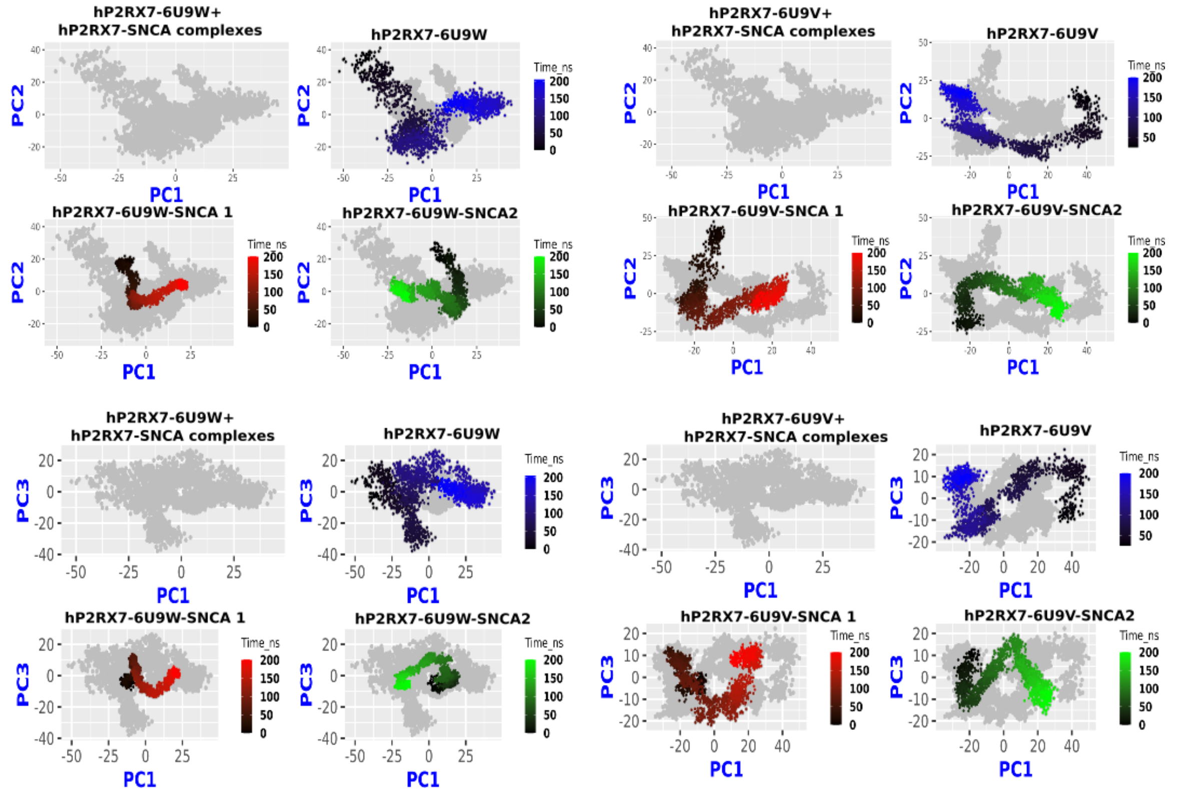

S2 Fig. Dynamical changes induced by αSyn on P2RX7 in larger trajectory.

(A) Backbone RMSD of αSyn alone, P2RX7 alone and P2RX7-αSyn complex of all the open and close forms of P2RX7 with and without αSyn were estimated for 200 ns time frame. The higher RMSD of αSyn alone is the distinguished in the line graph as compared to apoP2RX7 and P2RX7-SNCA complexes. The topmost left and right upper panel shows RMSD of closed (hP2RX7-6U9V) and open forms (hP2RX7-6U9W) of apoP2RX7 as orange line graph. The left and right middle panel shows RMSD of SNCA alone (blue), hP2RX7 alone (orange) and hP2RX7-SNCA complex (green) in line graph of respective closed and open forms of P2RX7-SNCA complex of replicate-1(hP2RX7-6U9V-SNCA1 & hP2RX7-6U9W-SNCA1). Similarly, the left and right bottom panel shows RMSD of SNCA alone (blue), hP2RX7 alone (orange) and hP2RX7-SNCA complex (green) in line graph of respective closed and open forms of P2RX7-SNCA complex of replicate-2 (hP2RX7-6U9V-SNCA2 & hP2RX7-6U9W-SNCA2). (B) The radius of gyration (Rg) analysis reveals that αSyn alone is less compact compared to both apo P2RX7 and the P2RX7-SNCA complexes. Interestingly, apo P2RX7 exhibits a slightly higher Rg than the protein complexes, indicating a marginally more compact structure. The color scheme used aligns with that of the RMSD analysis. (C) RMSF of apoP2RX7 and P2RX7-SNCA complex models after 200 ns MD simulations. There are certain regions in P2RX7 in P2RX7 in both apo and complexes which are quite flexible. Most of these regions (Head, ATP binding sites, cytoplasmic ballast). The open form of P2RX7 (hP2RX7-6U9W) is depicted in aquamarine, while the closed form (hP2RX7-6U9V) is shown in dark orange. Their respective complexes are represented as follows: hP2RX7-6U9W-SNCA1 and hP2RX7-6U9W-SNCA2 in sky blue and pink, and hP2RX7-6U9V-SNCA1 and hP2RX7-6U9V-SNCA2 in khaki and green. (D) Distribution of first 10 eigenvectors of all the models

after PCA analysis of 175 ns MD trajectories (25-200ns). First three eigenvectors of each model within the box show their proportion in the bar diagram labelled with eigenvector percentage analysis. The color schemes are same as followed in RMSF analysis. (E) Concatenated MD trajectory of respective apoP2RX7 and P2RX7-SNCA complexes of larger 175 ns MD simulations were used for PCA analysis. The projection of PC1 vs PC2, PC1 vs PC3, and PC2 vs PC3 for concatenated trajectories of apoP2RX7 and the P2RX7-αSyn (SNCA) complex using eigenvectors shows that certain subsets of conformations overlap in the apoP2RX7 and P2RX7-αSyn complexes, particularly in the central regions of the plots. This overlap suggests structural similarities between the apoP2RX7 and the P2RX7-αSyn complex. However, in the PC1 vs PC2 plot, isolated pockets are located away from the centre, indicating structural dissimilarities. This observation implies that while there are common structural features, some regions of the conformational space exhibit notable differences between the Apo and bound states. The colour codes are same as shown in Figure3C. (F) Time and space evolution of conformational space of individual MD trajectories of open and close forms of P2RX7-SNCA complexes for 200ns MD simulations. The eigenvectors were obtained by PCA analysis and there after projected the PC1 vs PC2 and PC1 vs PC3 in 2D space. The upper left and right panel shows PC1 vs PC2 of close (hP2RX7-6U9V) and open forms (hP2RX7-6U9W) of P2RX7 with their corresponding SNCA complexes (hP2RX7-6U9W_SNCA1/2 & hP2RX7-6U9V-SNCA1/2). The grey colour scattered plot shows the PC1vs PC2 of apoP2RX7 and all the SNCA complexes. The colour transition from black to blue (apoP2RX7), black to red blue (P2RX7-SNCA-Complex-1), and black to green (P2RX7-SNCA-Complex-2), indicates time progression from 0 to 200 timescales. Likewise, PC1 vs PC3 of open and close forms were shown in the lower left and right panel and were depicted by the same colour pattern in relation to different timeframes. The results show that conformational space of all the complexes are evolved well during in different time points of MD simulation. Both PC1 vs PC2 and PC1 vs PC3 shows the conformational space of apo P2RX7 close and open forms are evolved well and exist in different clusters showing distinct conformational evolutions during MD simulations. Likewise, P2RX7-SNCA complexes were also evolved well but less diversified with fewer clusters and progressively but narrowly undergoes different conformational transitions. The results demonstrate that the conformational space of all complexes evolves effectively at different time points during the MD simulation. The PCA plots (PC1 vs. PC2 and PC1 vs. PC3) reveal that both the closed and open forms of apo P2RX7 exhibit well-defined evolution of conformational space, existing in distinct clusters that show divergent conformational transitions throughout the simulation. In contrast, the P2RX7-SNCA complexes also exhibit significant conformational evolution; however, they are less diversified, with fewer clusters observed. These complexes undergo progressive, yet more narrowly confined, conformational transitions compared to the apo form, suggesting a more constrained dynamic range due to the binding of SNCA.
