# Supplementary material for: Mechanistic insights into Alpha-Synuclein binding to P2RX7: A molecular dynamic and docking study
Source: PLoS One. 2025 May 2;20(5):e0319098. doi: 10.1371/journal.pone.0319098 (PMC12047839; doi:10.1371/journal.pone.0319098)
Supplement: S3 Fig — (A) Perform a pairwise distance analysis for residues D435 and D438 in chain A, comparing these distances to the corresponding residues in chains B and C within both the apo and P2RX7-SNCA complexes using GROMACS software. After computing the distances, sum them separately for D435 and D438 and visualize the results with a line plot. (B) Illustrate the cytoplasmic pore structure, focusing on the α12- and α13-helices, and the cytoplasmic plug represented by the α9-helix, using cartoon diagrams. (C and D) Conduct radius of gyration (Rg) analysis for residues D435 and D438 in both the apo and P2RX7-SNCA complexes. (PDF) [file pone.0319098.s003.pdf]

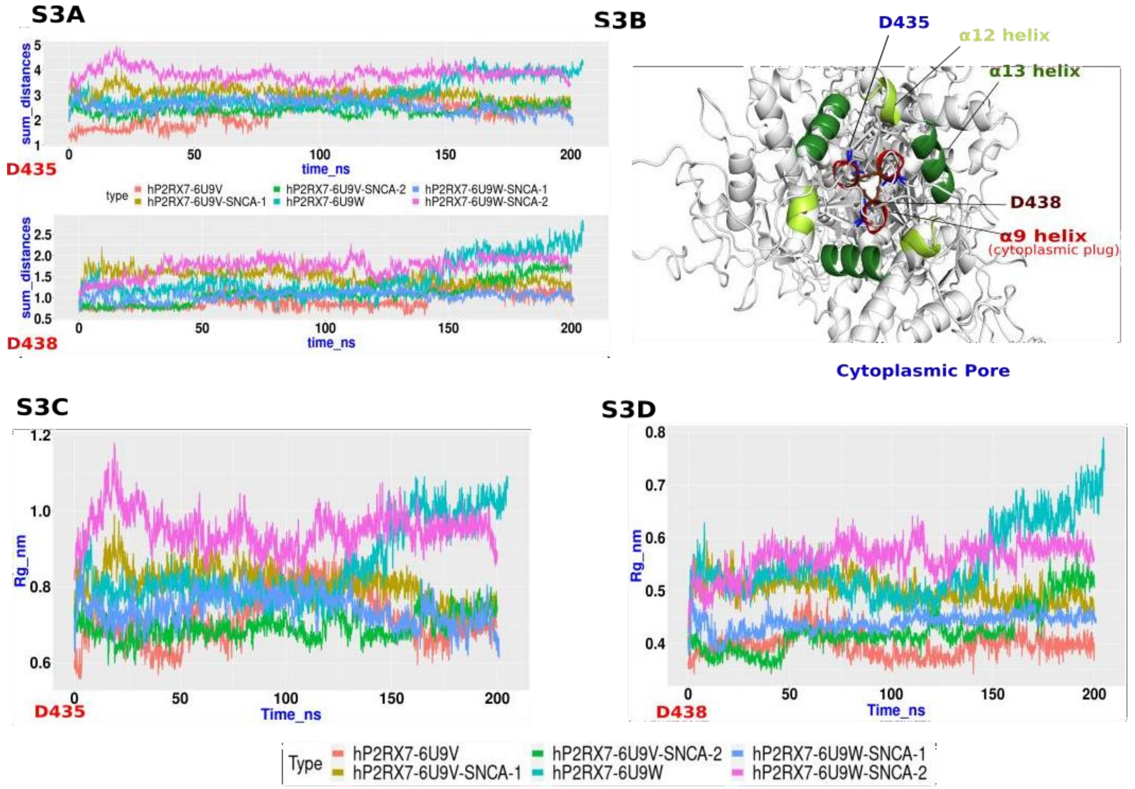

S3 Fig.Cytoplasmic pore dynamics.

(A) Perform a pairwise distance analysis for residues D435 and D438 in chain A, comparing these distances to the corresponding residues in chains B and C within both the apo and P2RX7-SNCA complexes using GROMACS software. After computing the distances, sum them separately for D435 and D438 and visualize the results with a line plot. (B) Illustrate the cytoplasmic pore structure, focusing on the α12- and α13-helices, and the cytoplasmic plug represented by the α9-helix, using cartoon diagrams. (C and D) Conduct radius of gyration (Rg) analysis for residues D435 and D438 in both the apo and P2RX7-SNCA complexes.
