# Supplementary material for: Mechanistic insights into Alpha-Synuclein binding to P2RX7: A molecular dynamic and docking study
Source: PLoS One. 2025 May 2;20(5):e0319098. doi: 10.1371/journal.pone.0319098 (PMC12047839; doi:10.1371/journal.pone.0319098)
Supplement: S4 Fig — The upper left and right panel shows secondary structural changes in open (hP2RX7-6U9W) and close (hP2RX7-6U9V) forms of apoP2RX7. The secondary structural changes in first replicate of hP2RX7-SNCA complexes of both open and close forms were shon in the middle left and right panel. Likewise, the bottom left and right panel shows second replicate of open and close forms of hP2RX7-SNCA complexes. In the figure, beta sheet, alpha helix, turn, 5-Helix, Bend and Beta Bridge are indicated by red, blue, yellow purple, green and dark grey colour whereas coil with no color. (PDF) [file pone.0319098.s004.pdf]

S4.

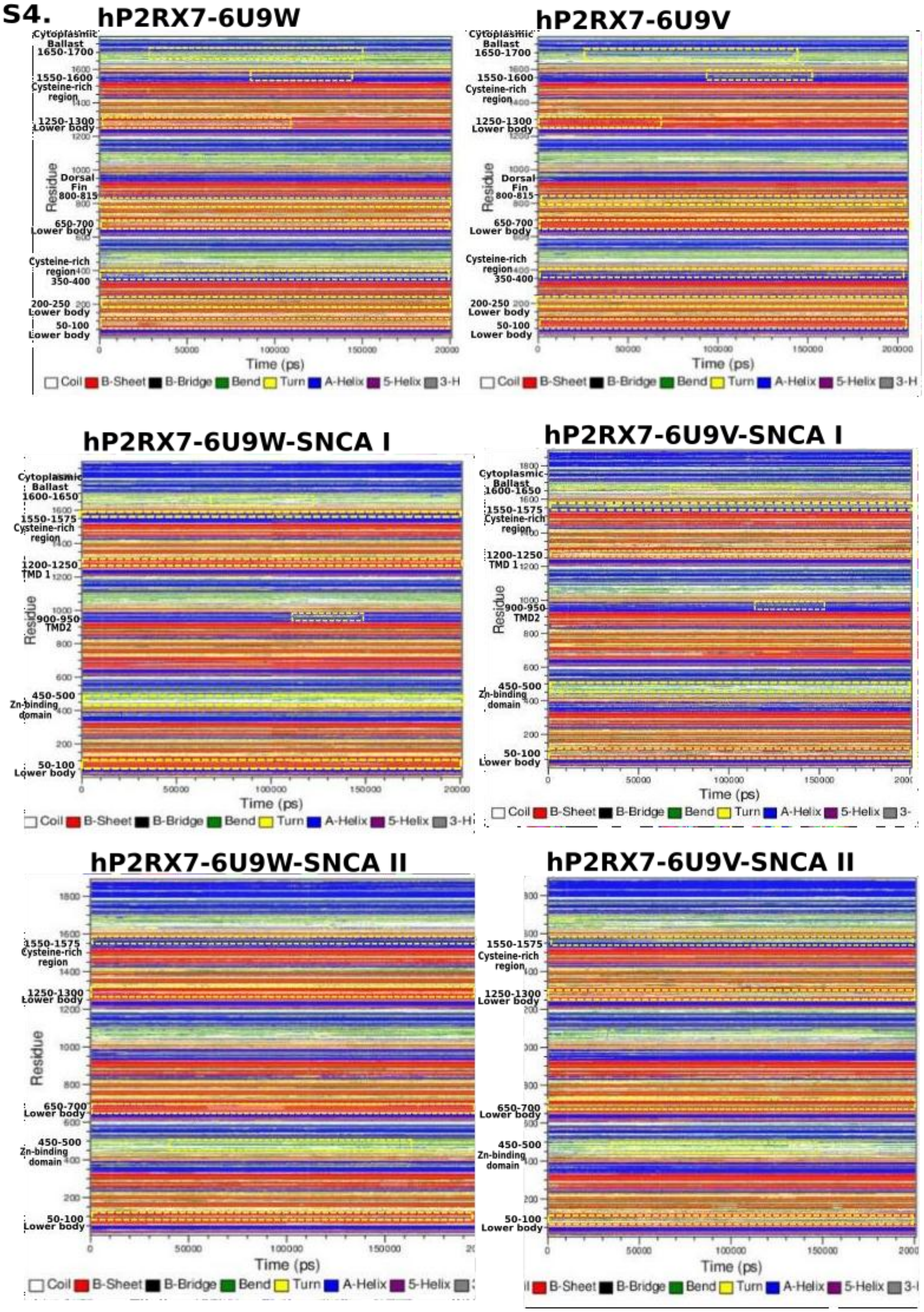

hP2RX7-6U9W
hP2RX7-6U9V
hP2RX7-6U9W-SNCA I
hP2RX7-6U9V-SNCA I
hP2RX7-6U9W-SNCA II
hP2RX7-6U9V-SNCA II
Cytoplasmic Ballast
1650-1700
1600-1650
1550-1600
1550-1575
Cysteine-rich region
1250-1300
1200-1250
Lower body
TMD 1
Dorsal Fin
800-815
900-950
TMD2
650-700
450-500
Zn-binding domain
350-400
200-250
50-100
Residue
Time (ps)
Coil
B-Sheet
B-Bridge
Bend
Turn
A-Helix
5-Helix
3-H

S4 Fig. Assessment of secondary structural changes in HP2RX7 upon α interaction by DSSP during 200ns MD simulations.

The upper left and right panel shows secondary structural changes in open (hP2RX7-6U9W) and close (hP2RX7-6U9V) forms of apoP2RX7. The secondary structural changes in first replicate of hP2RX7-SNCA complexes of both open and close forms were shown in the middle left and right panel. Likewise, the bottom left and right panel shows second replicate of open and close forms of hP2RX7-SNCA complexes. In the figure. beta sheet, alpha helix, turn, 5-Helix, Bend and Beta Bridge are indicated by red, blue, yellow purple, green and dark grey colour whereas coil with no colour.
