# Supplementary material for: Mechanistic insights into Alpha-Synuclein binding to P2RX7: A molecular dynamic and docking study
Source: PLoS One. 2025 May 2;20(5):e0319098. doi: 10.1371/journal.pone.0319098 (PMC12047839; doi:10.1371/journal.pone.0319098)
Supplement: S5 Fig — A) The Alpha-Beta Collective variables from the PLUMED plugin were used to assess the alpha and beta strand contents by analyzing standard phi and psi angles of -135° and 135° in both the apo and P2RX7-SNCA complexes. The results are displayed as a line plot. The graph shows in upper panel, the open forms of apoP2RX7 in dark orange while the corresponding SNCA complexes are shown in dark olive green hP2RX7- 6U9W- SNCA-1) and cornflower blue (hP2RX7- 6U9W- SNCA-2). The same colour code was depicted in lower panel for apo forms of closed P2RX7 and corresponding complexes (hP2RX7- 6U9V1- SNCA-1 and hP2RX7- 6U9V1- SNCA-2). B) Cartoon diagrams illustrate the cytoplasmic cap, which is composed of β-1, β0, and β15 beta strands, colour blue, maroon, and green, respectively. C) The α-helix content of the cytoplasmic pore (α12 and α13) and the cytoplasmic plug (α9) was calculated using αRMSD and presented as a line graph. The color code was same as shown in figure A. (PDF) [file pone.0319098.s005.pdf]

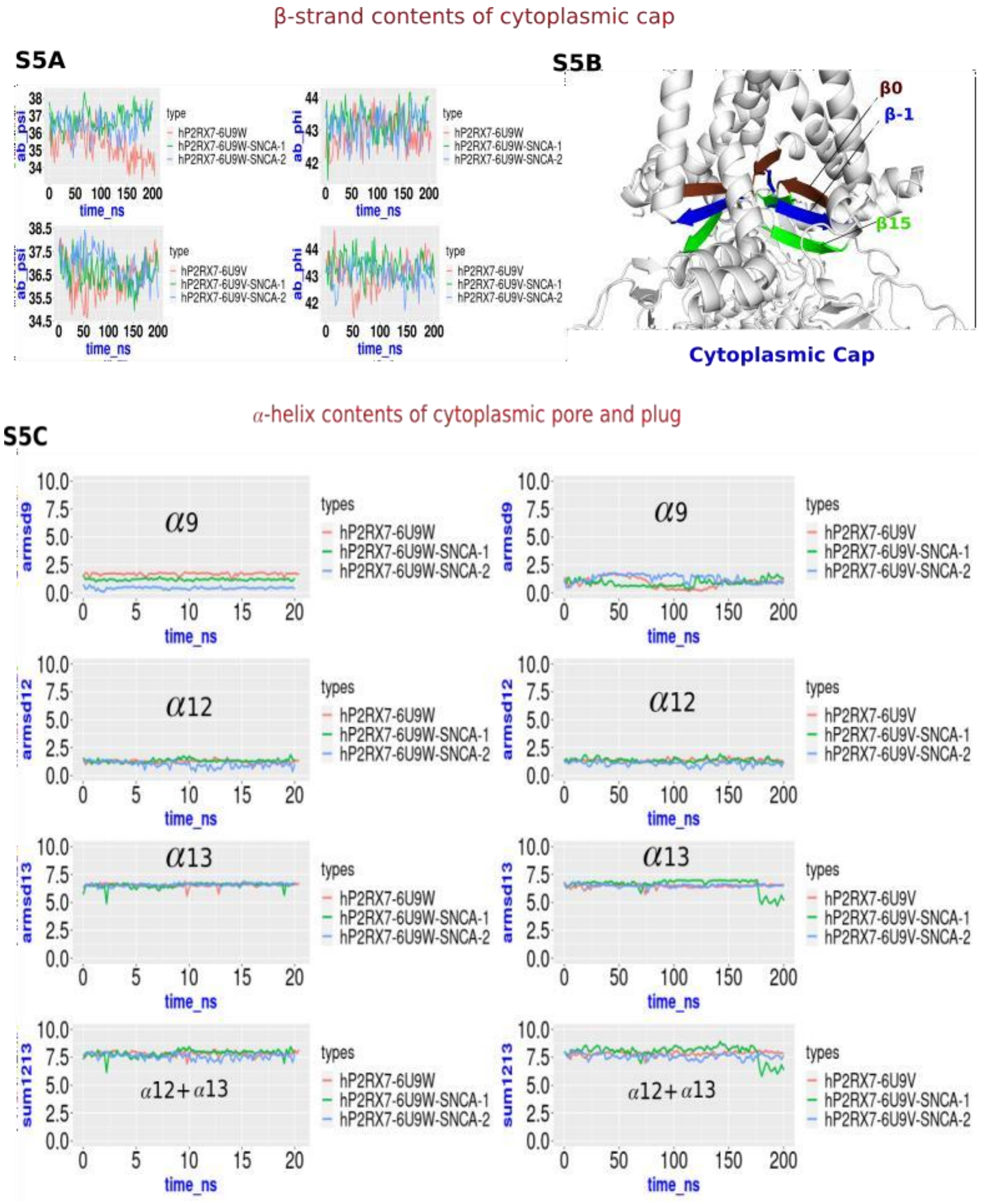

S5 Fig. The evaluation of cytoplasmic cap stability.

A) The Alpha-Beta Collective variables from the PLUMED plugin were used to assess the alpha and beta strand contents by analyzing standard phi and psi angles of -135° and 135° in both the apo and P2RX7-SNCA complexes. The results are displayed as a line plot. The graph shows in upper panel, the open forms of apoP2RX7 in dark orange while the corresponding SNCA complexes are shown in dark olive green hP2RX7- 6U9W- SNCA-1) and cornflower blue (hP2RX7- 6U9W- SNCA-2). The same colour code was depicted in lower panel for apo forms of closed P2RX7 and corresponding complexes (hP2RX7- 6U9V1- SNCA-1 and hP2RX7- 6U9V1- SNCA-2). B) Cartoon diagrams illustrate the cytoplasmic cap, which is

composed of β-1, β0, and β15 beta strands, colour blue, maroon, , and green, respectively. C) The α-helix content of the cytoplasmic pore (α12 and α13) and the cytoplasmic plug (α9) was calculated using αRMSD and presented as a line graph. The color code was same as shown in Figure A.
