# Supplementary material for: Mechanistic insights into Alpha-Synuclein binding to P2RX7: A molecular dynamic and docking study
Source: PLoS One. 2025 May 2;20(5):e0319098. doi: 10.1371/journal.pone.0319098 (PMC12047839; doi:10.1371/journal.pone.0319098)
Supplement: S6 Fig — A) The minimum distance analysis between α-Syn and P2RX7 showing quite close proximity. The results are displayed as a line plot. The graph shows corresponding P2RX7-SNCA complexes are shown in dark orange (hP2RX7- 6U9V- SNCA-1), dark olive green (hP2RX7- 6U9V- SNCA-2), corn blue (hP2RX7- 6U9W- SNCA-1) and purple (hP2RX7- 6U9W- SNCA-2). B) Minimum distances of each residues of P2RX7 to the α-Syn shows certain region of P2RX7 quite close to the α-Syn, most of these regions are trans membrane domains in chain A and chain B. The same colour codes of legend were followed shown in previous figure. C) The contact analysis by CONAN using 5Å distance cut-off shows persistence of contacts and number of contact formation. D) Solvent Accessibility Surface Area (SASA) analysis of P2RX7 residues shows proximity to the α-Syn. Most of the residues show higher solvent accessibility. The graph in left panel shows the open forms of apoP2RX7 in dark orange while the corresponding SNCA complexes are shown in dark olive green hP2RX7- 6U9W- SNCA-1) and cornflower blue (hP2RX7- 6U9W- SNCA-2). The same colour code was depicted in right panel for apo forms of closed P2RX7 and corresponding complexes (hP2RX7- 6U9V1- SNCA-1 and hP2RX7- 6U9V1- SNCA-2). (PDF) [file pone.0319098.s006.pdf]

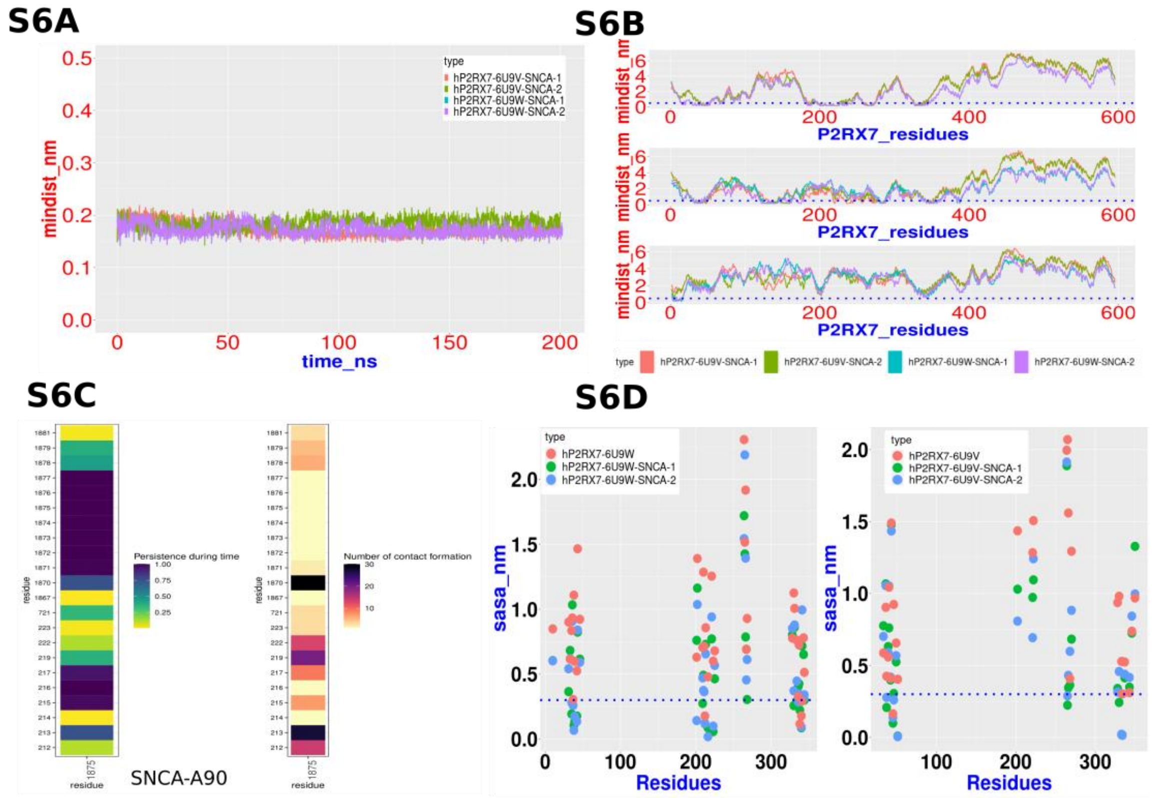

S6 Fig. The evaluation of contacts between P2RX7 and α-Syn.

A) The minimum distance analysis between α-Syn and P2RX7 showing quite close proximity. The results are displayed as a line plot. The graph shows corresponding P2RX7-SNCA complexes are shown in dark orange (hP2RX7-6U9V- SNCA-1), dark olive green (hP2RX7- 6U9V- SNCA-2), corn blue (hP2RX7- 6U9W- SNCA-1) and purple (hP2RX7- 6U9W- SNCA-2). B) Minimum distances of each residues of P2RX7 to the α-Syn shows certain region of P2RX7 quite close to the α-Syn, most of these regions are trans membrane domains in chain A and chain B. The same colour codes of legend were followed shown in previous Figure. C) The contact analysis by CONAN using 5Å distance cut-off shows persistence of contacts and number of contact formation. D) Solvent Accessibility Surface Area (SASA) analysis of P2RX7 residues shows proximity to the α-Syn. Most of the residues show higher solvent accessibility**.** The graph in left panel shows the open forms of apoP2RX7 in dark orange while the corresponding SNCA complexes are shown in dark olive green hP2RX7-6U9W- SNCA-1) and cornflower blue (hP2RX7- 6U9W- SNCA-2). The same colour code was depicted in right panel for apo forms of closed P2RX7 and corresponding complexes (hP2RX7- 6U9V1- SNCA-1 and hP2RX7- 6U9V1- SNCA-2).
