# Supplementary material for: Mechanistic insights into Alpha-Synuclein binding to P2RX7: A molecular dynamic and docking study
Source: PLoS One. 2025 May 2;20(5):e0319098. doi: 10.1371/journal.pone.0319098 (PMC12047839; doi:10.1371/journal.pone.0319098)
Supplement: S7 Fig — (A) The number of hub residues identified from the PSN analysis of P2RX7-SNCA complexes, specifically hP2RX7-6U9W-SNCA and hP2RX7-6U9V-SNCA. The PSN was constructed using contact information derived from salt bridge interactions (cut-off 4.5 Å), hydrogen bonds (cut-off 3.5 Å), and hydrophobic contacts (cut-off 5 Å) obtained from MD trajectories. These hub residues are crucial for maintaining the structural integrity and function of the protein complex. (B) The top 5 connected components identified from the PSN analysis of the hP2RX7-6U9W-SNCA and hP2RX7-6U9V-SNCA complexes. These components represent the most interconnected and stable regions of the protein structure during the 200 ns MD simulations, indicating key regions of structural and functional significance within the complex. (PDF) [file pone.0319098.s007.pdf]

S7A

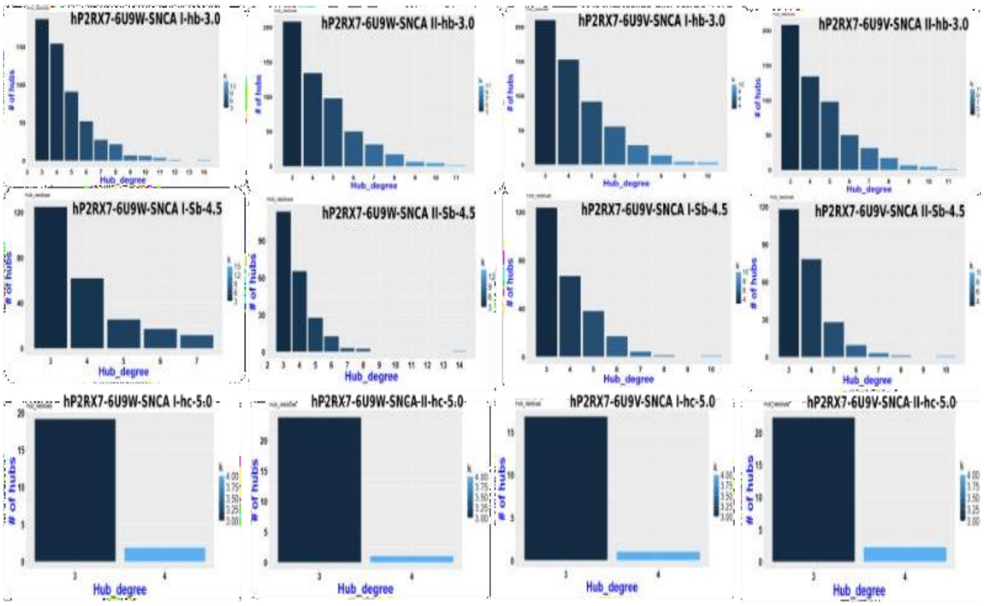

S7B

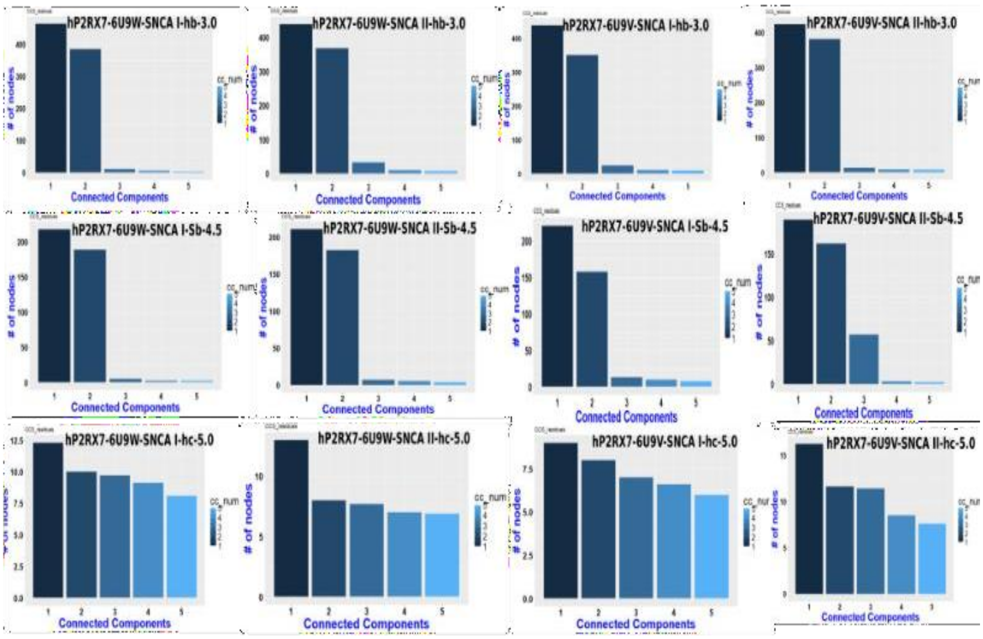

S7 Fig. Hub and connected component analysis of P2RX7-SNCA Complex using Protein Structure Network (PSN) Analysis.

(A) The number of hub residues identified from the PSN analysis of P2RX7-SNCA complexes, specifically hP2RX7-6U9W-SNCA and hP2RX7-6U9V-SNCA. The PSN was constructed using contact information derived from salt bridge interactions (cut-off 4.5 Å), hydrogen bonds (cut-off 3.5 Å), and hydrophobic contacts (cut-off 5 Å) obtained from MD trajectories. These hub residues are crucial for maintaining the structural integrity and function of the protein complex. (B) The top 5 connected components identified from the PSN analysis of the hP2RX7-6U9W-SNCA and hP2RX7-6U9V-SNCA complexes. These components represent the most interconnected and stable regions of the protein structure during the 200 ns MD simulations, indicating key regions of structural and functional significance within the complex.
