# Supplementary material for: Mechanistic insights into Alpha-Synuclein binding to P2RX7: A molecular dynamic and docking study
Source: PLoS One. 2025 May 2;20(5):e0319098. doi: 10.1371/journal.pone.0319098 (PMC12047839; doi:10.1371/journal.pone.0319098)
Supplement: Attachment — Submitted filename: Response_to_reviewver.docx [file pone.0319098.s017.docx]

**Points raised by the academic editor and reviewer(s)**

**1**. Please ensure that your manuscript meets PLOS ONE's style requirements, including those for file naming. The PLOS ONE style templates can be found at [https://journals.plos.org/plosone/s/file?id=wjVg/PLOSOne_formatting_sample_main_body.pdf](https://track.editorialmanager.com/CL0/https:%2F%2Fjournals.plos.org%2Fplosone%2Fs%2Ffile%3Fid=wjVg%2FPLOSOne_formatting_sample_main_body.pdf/1/010f0193217a29de-cda273cc-9e2f-46e2-a2a3-ff91866e841c-000000/6DIOwklJGHNFysj68k-_zEuMHE1G4jDOmLOTSzZO3FY=184) and[https://journals.plos.org/plosone/s/file?id=ba62/PLOSOne_formatting_sample_title_authors_affiliations.pdf](https://track.editorialmanager.com/CL0/https:%2F%2Fjournals.plos.org%2Fplosone%2Fs%2Ffile%3Fid=ba62%2FPLOSOne_formatting_sample_title_authors_affiliations.pdf/1/010f0193217a29de-cda273cc-9e2f-46e2-a2a3-ff91866e841c-000000/RLSvHujv9bsyuEGrk2b-Uw0_IPkSuYj_YfzfpwZOtbA=184)

**Response:** We followed the standard template style including “Level 1 heading for all major sections (Abstract, Introduction, Materials and methods, Results, Discussion,Acknowledgment and References.)”, “Bold type”, “18pt font”. Headings are written in sentence case (capitalize only the first word of the heading, the first word of the subheading, and any proper nouns and genus names). The Figures are Cited as “Fig 1”, “Fig 2” and tables are cited as Table1. Supplementary figures and tables are cited as S1A Fig. If multiple figures are required to cite in one place, then cited as Fig1A-B, S1A-D etc. Each figure caption appears directly after the paragraph in which they are first cited. We used bold type for the figure titles. We used Level 2 headings for sub-sections of major sections.

2. Please update your submission to use the PLOS LaTeX template. The template and more information on our requirements for LaTeX submissions can be found at http://[journals.plos.org/plosone/s/latex](http://journals.plos.org/plosone/s/latex).

**Response:** We followed the http://[journals.plos.org/plosone/s/latex](http://journals.plos.org/plosone/s/latex) for implementation of LaTeX template styles in our manuscript.

**3.** Please note that PLOS ONE has specific guidelines on code sharing for submissions in which author-generated code underpins the findings in the manuscript. In these cases, we expect all author-generated code to be made available without restrictions upon publication of the work. Please review our guidelines at https://journals.plos.org/plosone/s/materials-and-software-sharing # loc-sharing-code and ensure that your code is shared in a way that follows best practice and facilitates reproducibility and reuse.

**Response:** All the raw data and codes of present study are shared in <https://figshare.com/account/items/28152182/edit>,<https://github.com/MukDAN/IVRI_P2RX7_SNCA_DATA> and <https://zenodo.org/uploads/13710700> repositories. The old data archived in Zenedo (<https://zenodo.org/me/requests/b5cf259a-623c-4087-81a9-4568902df37e>) has embargo upto 31/01/2025. These data and codes are freely available for access publicly.

**4. Thank you for stating the following financial disclosure:**

“Mukesh Kumar

No.F.7-40/Biochem./2022-23/JD(R))

ICAR-Indian Veterinary Research Institute

https://www.ivri.nic.in/

No”

Please state what role the funders took in the study. If the funders had no role, please state: "The funders had no role in study design, data collection and analysis, decision to publish, or preparation of the manuscript."

If this statement is not correct you must amend it as needed.

Please include this amended Role of Funder statement in your cover letter; we will change the online submission form on your behalf.

**Response:**  The statement "The funders had no role in study design, data collection and analysis, decision to publish, or preparation of the manuscript",has been added during revised submission.

**5. Thank you for stating the following in the Acknowledgments Section of your manuscript:“Our present manuscript is supported by the institute funded project entitled “Elucidating the role of alpha synuclein (SNCA) and cholesterol on regulating the P2RX7 pore dilation”No.F.740/Biochem./2022-23/JD(R). We thank the Director, ICAR-IVRI and Joint DirectorResearch ICAR-IVRI for their kind support. We thank the head, Division of Biochemistry for his valuable support and cooperation to execute the research project. We would like to thank the faculty of Biochemistry, Dr. Praveen Singh, Dr. Ajay Kumar and Dr. Meeta Saxena for their valuable guidance and useful feedback. We thanked technical officer Puja and attendant Riyasat Ji for his valuable support during conducting research in the lab. We would like to thank Dr.Himanshu Khandelia, Asso. Professor, South Denmark University, Denmark for his valuableSuggestions during MD data analysis.”**

**We note that you have provided funding information that is currently declared in your Funding Statement. However, funding information should not appear in the Acknowledgments section or other areas of your manuscript. We will only publish funding information present in the Funding Statement section of the online submission form.Please remove any funding-related text from the manuscript and let us know how you would like to update your Funding Statement. Currently, your Funding Statement reads as follows:**

“Mukesh Kumar

No.F.7-40/Biochem./2022-23/JD(R))

ICAR-Indian Veterinary Research Institute

https://www.ivri.nic.in/

No”

Please include your amended statements within your cover letter; we will change the online submission form on your behalf.

**Response:** The funding related text is removed from the acknowledgement section of manuscript and we have updated in the same way.

6. When completing the data availability statement of the submission form, you indicated that you will make your data available on acceptance. We strongly recommend all authors decide on a data sharing plan before acceptance, as the process can be lengthy and hold up publication timelines. Please note that, though access restrictions are acceptable now, your entire data will need to be made freely accessible if your manuscript is accepted for publication. This policy applies to all data except where public deposition would breach compliance with the protocol approved by your research ethics board. If you are unable to adhere to our open data policy, please kindly revise your statement to explain your reasoning and we will seek the editor's input on an exemption. Please be assured that, once you have provided your new statement, the assessment of your exemption will not hold up the peer review process.

**Response:** All the raw data and codes of present study are shared in <https://figshare.com/account/items/28152182/edit>,<https://github.com/MukDAN/IVRI_P2RX7_SNCA_DATA> and <https://zenodo.org/uploads/13710700> repositories. The old data archived in Zenedo (<https://zenodo.org/me/requests/b5cf259a-623c-4087-81a9-4568902df37e>) has embargo upto 31/01/2025. These data and codes are freely available for access publicly without restriction.

**7. We note that you have included the phrase “data not shown” in your manuscript. Unfortunately, this does not meet our data sharing requirements. PLOS does not permit references to inaccessible data. We require that authors provide all relevant data within the paper, Supporting Information files, or in an acceptable, public repository. Please add a citation to support this phrase or upload the data that corresponds with these findings to a stable repository (such as Figshare or Dryad) and provide URLs, DOIs, or accession numbers that may be used to access these data. Or, if the data are not a core part of the research being presented in your study, we ask that you remove the phrase that refers to these data.**

**Response :** The phrase “data not shown” in the manuscript, is removed and the respective information is replaced by the supplementary figure S2A in manuscript. All the raw data and codes are freely available to <https://figshare.com/account/items/28152182/edit>,<https://github.com/MukDAN/IVRI_P2RX7_SNCA_DATA>and <https://zenodo.org/uploads/13710700> repositories. The old data archived in Zenedo (<https://zenodo.org/me/requests/b5cf259a-623c-4087-81a9-4568902df37e>)

**Additional Editor Comments:**

The submission has an ideal scientific significance, however, needs to be revised qualitatively to meet the standard for publication in this journal.

[Note: HTML markup is below. Please do not edit.]

Reviewers' comments:

Reviewer's Responses to Questions

**Comments to the Author**

**1. Is the manuscript technically sound, and does the data support the conclusions?**

The manuscript must describe a technically sound piece of scientific research with data that supports the conclusions. Experiments must have been conducted rigorously, with appropriate controls, replication, and sample sizes. The conclusions must be drawn appropriately based on the data presented.

Reviewer #1: Yes

Reviewer #2: Yes

Reviewer #3: Yes

**2. Has the statistical analysis been performed appropriately and rigorously?**

Reviewer #1: I Don't Know

Reviewer #2: Yes

Reviewer #3: Yes

**3. Have the authors made all data underlying the findings in their manuscript fully available?**

The [PLOS Data policy](http://track.editorialmanager.com/CL0/http:%2F%2Fwww.plosone.org%2Fstatic%2Fpolicies.action%23sharing/1/010f0193217a29de-cda273cc-9e2f-46e2-a2a3-ff91866e841c-000000/JzRN-xOxQlMhBfMzKQu5Okiy9jGhq1MzjJx7r5QLAz0=184) requires authors to make all data underlying the findings described in their manuscript fully available without restriction, with rare exception (please refer to the Data Availability Statement in the manuscript PDF file). The data should be provided as part of the manuscript or its supporting information, or deposited to a public repository. For example, in addition to summary statistics, the data points behind means, medians and variance measures should be available. If there are restrictions on publicly sharing data—e.g. participant privacy or use of data from a third party—those must be specified.

**Reviewer #1: No**

**Reviewer #2: Yes**

**Reviewer #3: Yes**

**4. Is the manuscript presented in an intelligible fashion and written in standard English?**

PLOS ONE does not copy edit accepted manuscripts, so the language in submitted articles must be clear, correct, and unambiguous. Any typographical or grammatical errors should be corrected at revision, so please note any specific errors here.

**Reviewer #1: Yes**

**Reviewer #2: Yes**

**Reviewer #3: Yes**

**5. Review Comments to the Author**

Please use the space provided to explain your answers to the questions above. You may also include additional comments for the author, including concerns about dual publication, research ethics, or publication ethics. (Please upload your review as an attachment if it exceeds 20,000 characters)

**Reviewer #1: The manuscript is solid, and the hypothesis is well-defined and supported by preliminary evidence. However, some underlying evidence obtained by the author needed to be provided in the manuscript, which negates the journal guideline. For instance, variation in the RMSD of ⍺Syn was not shown. Additionally, data on the spatiotemporal evolution of each protein was not given.**

**Response:**  We have addressed the reviewer’s suggestions and made the necessary corrections following the journal's guidelines. The reviewer’s concern regarding the RMSD of ⍺Syn in individual complexes is addressed in the supplementary figure S2A. This figure also allows for an analysis of ⍺Syn, P2RX7, and P2RX7-SNCA complexes from the perspective of time and spatial evolution. Spatio-temporal evolution of the molecular system can be explained by RMSD, RMSF, Radius of gyration, secondary structure analysis and PCA. Already we have shown RMSD (Fig 3A), RMSF (Figure S2C), PCA ( Fig 3C & S2E)) and Secondary structure analysis (Fig S4) of each protein which can indicate the spatio-temporal evolution of conformational space. However, apart from that we have again added **RMSD (Fig S2A), Radius of gyration (Rg) (Fig S2B), Conformational dynamics (PC1 vs PC2 and PC1 vs PC3 projection in 2D graphs) of individual trajectories for 200 ns timescales (Fig S2F)** in order to show the time and space evolutions of conformational space of individual P2RX7-SNCA complexes.

**In the images below , we have described the newly added figures and graphs (Fig S2A-B & Fig S2F).**

**Supplementary Fig S2A:** The figure shows the backbone RMSD of 𝛼Syn alone, P2RX7 alone and P2RX7-𝛼Syn complex of all the open and close forms of P2RX7 with and without 𝛼Syn for 200 ns time frame. The X axis is denoting the time (in ns) and the y axis is RMSD (in nm). The higher RMSD of 𝛼Syn alone is the distinguished in the line graph as compared to apoP2RX7 and P2RX7-SNCA complexes. The topmost left and right upper panel shows RMSD of closed (hP2RX7-6U9V) and open forms (hP2RX7-6U9W) of apoP2RX7 as orange line graphs. The left and right middle panel shows RMSD of SNCA alone (blue), hP2RX7 alone (orange) and hP2RX7-SNCA complex (green) in line graph of respective closed and open forms of P2RX7-SNCA complex of replicate-1(hP2RX7-6U9V-SNCA1 & hP2RX7-6U9W-SNCA1). Similarly, the left and right bottom panel shows RMSD of SNCA alone (blue), hP2RX7 alone (orange) and hP2RX7-SNCA complex (green) in line graph of respective closed and open forms of P2RX7-SNCA complex of replicate-2 (hP2RX7-6U9V-SNCA2 & hP2RX7-6U9W-SNCA2).

**Supplementary Fig S2B: The figure shows** the radius of gyration (Rg) that reveals that compactness of α-synuclein (αSyn) alone is very poor as compared to both apo P2RX7 and the P2RX7-SNCA complexes. Interestingly, apo P2RX7 exhibits a slightly higher Rg than the protein complexes, indicating a marginally more compact structure probably due to the effects of α-synuclein. The color scheme used aligns with that of the RMSD analysis.

**Supplementary Fig S2F:** Time and space evolution of conformational space of individual MD trajectories of open and close forms of apoP2RX7 and P2RX7-SNCA complexes for 200ns MD simulations. The eigenvectors were obtained by PCA analysis and thereafter projected the PC1 vs PC2 and PC1 vs PC3 in 2D space. The upper left and right panel shows PC1 vs PC2 of close (hP2RX7-6U9V) and open forms (hP2RX7-6U9W) of P2RX7 with their corresponding SNCA complexes (hP2RX7-6U9W_SNCA1/2 & hP2RX7-6U9V-SNCA1/2). The grey colour scatter plot shows the PC1vs PC2 of apoP2RX7 and all the SNCA complexes. The colour transition from black to blue (apoP2RX7), black to red blue (P2RX7-SNCA-Complex-1), and black to green (P2RX7-SNCA-Complex-2), indicates time progression from 0 to 200 timescales. Likewise, PC1 vs PC3 of open and close forms were shown in the lower left and right panel and were depicted by the same colour pattern in relation to different timeframes. The results show that conformational space of all the complexes evolved well during different time points of MD simulation. Both PC1 vs PC2 and PC1 vs PC3 shows the conformational space of apo P2RX7 close and open forms are evolved well and exist in different clusters showing distinct conformational evolutions during MD simulations. Likewise, P2RX7-SNCA complexes were also evolved well but less diversified with fewer clusters and progressively but narrowly underwent different conformational transitions. The results demonstrate that the conformational space of all complexes evolves effectively at different time points during the MD simulation.

**Review-1 comments within manuscript contents**: All the highlighted concerns and suggestions shown in the manuscript contents by reviewer-1 are rectified, supplemented with respective information and shown as track change and markup.

**Reviewer #2: The article is a very good one which will open the way in managing neurodegenerative diseases in the future. The author was able to clearly explain the molecular interaction between P2RX7 and Syn in terms of structural pore intracellularly and extracellularly.**

**However, the article would be much better by having a table of content. Also, the method section ----- structure analysis should be changed to structural analysis.**

**Response:** We have created the Table of contents, but could not find the right place in the manuscript to keep it. The structure analysis in the method section is replaced by “Structural analysis”.

| **Table of contents** | |
| --- | --- |
| **Abstract** | **1-2** |
| **Author summary** | **2** |
| **Introduction** | **2-5** |
| **Material Method:** | **5-9** |
| **Results** | **9-34** |
| **Discussion** | **34-40** |
| **Acknowledgement** | **41** |
| **Reference** | **41-51** |
| **Supplementary information** | **51** |

**Reviewer #3: This is a good manuscript that is well written, However, the authors should endeavor to correct the little errors identified and highlighted below.**

**- The title is too generic, and may discourage readers, as this will be mistaken for another docking and attractive study, the problem the research addressed should be factor into the Title to make it more attractive and appealing.**

**Response:** The title of the research article is changed into “Mechanistic insights into Alpha-Synuclein binding to P2RX7: A molecular dynamics and docking Study” .

**- Spacing in between words and numbers such as which84, and24 spaced as “which 84”, as seen in 19**

**Response:** The typographical mistake was resolved.

**6. PLOS authors have the option to publish the peer review history of their article (**[**what does this mean?**](https://track.editorialmanager.com/CL0/https:%2F%2Fjournals.plos.org%2Fplosone%2Fs%2Feditorial-and-peer-review-process%23loc-peer-review-history/1/010f0193217a29de-cda273cc-9e2f-46e2-a2a3-ff91866e841c-000000/ZCcu--_FJFHhtSH36kgVTsUwtCJQ0j9ojEOfrQ2fNMg=184)**). If published, this will include your full peer review and any attached files.**

**If you choose “no”, your identity will remain anonymous but your review may still be made public.**

**Do you want your identity to be public for this peer review? For information about this choice, including consent withdrawal, please see our** [**Privacy Policy**](https://track.editorialmanager.com/CL0/https:%2F%2Fwww.plos.org%2Fprivacy-policy/1/010f0193217a29de-cda273cc-9e2f-46e2-a2a3-ff91866e841c-000000/ADJNoPEO4GJPOVJNAP_FZa2xcOEnR99Q_fXyn0RNw3w=184)**.**

**Reviewer #1: No**

**Reviewer #2: No**

**Reviewer #3: Yes: Sulyman Olalekan Ibrahim**
