# Supplementary material for: Mechanistic insights into Alpha-Synuclein binding to P2RX7: A molecular dynamic and docking study
Source: PLoS One. 2025 May 2;20(5):e0319098. doi: 10.1371/journal.pone.0319098 (PMC12047839; doi:10.1371/journal.pone.0319098)
Supplement: Attachment — Submitted filename: Reviewers Comment Plus one.docx [file pone.0319098.s013.docx]

This is a good manuscript that is well written, However, the authors should endeavor to correct the little errors identified and highlighted below.

- The title is too generic, and may discourage readers, as this will be mistaken for another docking and attractive study, the problem the research addressed should be factor into the Title to make it more attractive and appealing.
- Spacing in between words and numbers such as which84, and24 spaced as “which 84”, as seen in 19
- Asides these two minor observations, I found the manuscript to be well written, and detailly explained, I therefore recommend acceptance with minor revision to the title and the joint words and numbers
