# Supplementary material for: Mechanistic insights into Alpha-Synuclein binding to P2RX7: A molecular dynamic and docking study
Source: PLoS One. 2025 May 2;20(5):e0319098. doi: 10.1371/journal.pone.0319098 (PMC12047839; doi:10.1371/journal.pone.0319098)
Supplement: Attachment — Submitted filename: Recommendation-Plus one.docx [file pone.0319098.s015.docx]

Recommendation: Accept
